# Schmallenberg Virus as Possible Ancestor of Shamonda Virus

**DOI:** 10.3201/eid1810.120835

**Published:** 2012-10

**Authors:** Katja V. Goller, Dirk Höper, Horst Schirrmeier, Thomas C. Mettenleiter, Martin Beer

**Affiliations:** Friedrich-Loeffler-Institut, Greifswald-Insel Riems, Germany

**Keywords:** Schmallenberg virus, taxonomic classification, phylogeny, orthobunyavirus, Simbu serogroup, viruses, Shamonda virus, taxonomy

## Abstract

Schmallenberg virus (SBV), an orthobunyavirus of the Simbu serogroup, recently emerged in Europe and has been suggested to be a Shamonda/Sathuperi virus reassortant. Results of full-genome and serologic investigations indicate that SBV belongs to the species *Sathuperi virus* and is a possible ancestor of the reassortant Shamonda virus.

A novel virus, Schmallenberg virus (SBV), was discovered in Europe in October 2011, and since then, cases of SBV infection have been reported in sheep, cattle, and goats in several European countries (*1**–**4*). Preliminary phylogenetic analyses revealed that SBV is a member of the genus *Orthobunyavirus* within the family *Bunyaviridae* and is related to Simbu serogroup viruses (*1*). Similar to Akabane virus (AKAV), another Simbu serogroup virus, SBV can cause fatal congenital defects by infection of fetuses during a susceptible stage in pregnancy (*2*). Vaccines for SBV are not available. Thus, SBV poses a serious threat to naive populations of ruminant livestock in Europe.

Orthobunyaviruses are arthropod-borne viruses with a negative-stranded tripartite RNA genome comprising large (L), medium (M), and small (S) segments. Genetic reassortment occurs naturally among these viruses, which results in the emergence of new virus strains that have altered biologic properties (*5*). The L segment encodes the RNA-dependent RNA polymerase; antigenic determinants are the M-encoded viral surface glycoproteins Gn and Gc, which are responsible for viral attachment, cell fusion, hemagglutination, and the induction of neutralizing antibodies, and the S-encoded nucleocapsid protein N, which plays a role in complement fixation (*6*). In the pregenomics era, orthobunyavirus relationships were determined solely by serologic cross-reactivity analyses (*7*), but since DNA sequencing became available, phylogenetic relationships have additionally been assessed by comparison of partial genome sequences (*8**,**9*). However, published full-length genome sequence information is sparse, which makes in-depth phylogenetic analysis difficult. Therefore, a detailed taxonomic classification of SBV could not be made initially when the virus emerged.

The first report of SBV showed highest similarities of M- and L-segment sequences to partial Aino virus and AKAV sequences, whereas the N gene was most closely related to Shamonda virus (SHAV) (*1*). Additionally, results of recent investigations on complete N and M genes and partial L genes of SHAV, Douglas virus (DOUV), and Sathuperi virus (SATV) suggested that SBV is a reassortant consisting of the M segment from SATV and the S and L segments from SHAV (*9*). Conversely, in 2001, SHAV was described as a reassortant virus comprising the S and L segments of SATV and the M segment from the unclassified Yaba-7 virus (*8*). To clarify the phylogenetic relationships and classification of SBV within the Simbu serogroup, we conducted genetic and serologic investigations of its relationship to 9 other Simbu serogroup viruses.

## The Study

To enable comparative sequence analysis and phylogenetic investigations, we determined almost full-length S-, M-, and L-segment sequences for 9 Simbu serogroup viruses belonging to 5 species (Table 1): SHAV, Peaton virus, and Sango virus, species *Shamonda virus*; DOUV and SATV, species *Sathuperi virus*; Aino virus and Shuni virus, species *Shuni virus*; Sabo virus, species *Akabane virus*; and Simbu virus, species *Simbu virus*. Sample preparation and sequencing were done by using the Genome Sequencer FLX (Roche, Mannheim, Germany) as described (*10*). Sequence data obtained in this study are archived in the International Nucleotide Sequence Database Collaboration databases (www.insdc.org; accession nos. HE795087–HE795110 and HE800141–HE800143). In addition to the newly determined sequences, published full-genome sequences of AKAV and Oropouche virus (OROV) from the National Center for Biotechnology Information reference genome database (www.ncbi.nlm.nih.gov/sites/genome) were used for sequence comparisons and the reconstruction of phylogenetic relationships. Coding sequences of each genome segment were aligned by using ClustalW (www.clustal.org) for codons, and phylogenetic analyses were performed by using maximum-likelihood methods in MEGA5 (*11*). For the N and L gene analysis Tamura-Nei parameter, the M gene analysis Tamura 3-parameter was used. The robustness of the trees was tested by bootstrap analysis by using 1,000 replications. Sequence identities were calculated by using BioEdit version 7.0.9.0 (*12*).

**Table 1:** Viruses, isolates, and sequence lengths used in classification of Schmallenberg virus within the Simbu serogroup

Virus	Isolate	Sequence length, nt		
		S	M	L
Aino	38K	834	4,335	6,966
Douglas	93–6	813	4,365	6,857
Peaton	CSIRO 110	851	4,324	6,829
Sabo	IB AN 9398	894	4,307	6,857
Sango	An 5077	838	4,314	6,828
Sathuperi	NA	843	4,330	6,861
Schmallenberg	BH80/11–4	830	4,415	6,864
Shamonda	Ib An 5550	927	4,314	6,863
Shuni	Ib An 10107	850	4,326	6,880
Simbu	SA Ar 53	860	4,417	6,895

*S, small segment; M, medium segment; L, large segment; NA, not available.

SBV N gene nucleotide sequence identities to other viruses ranged from 69.8% (OROV; 69.9% aa identity) to 97.7% (SHAV; 100% aa) (Table 2). The L gene sequence of SBV had the lowest identity to OROV (60.4% nt; 57.5% aa) and highest identity to SHAV (92.9% nt; 98.4% aa); the SBV M gene showed the highest sequence identity to SATV (82.1% nt; 90.1% aa), whereas identity of SBV and SHAV M gene was low (48.2% nt; 36.5% aa). In general, identity of the SHAV M gene to the other Simbu serogroup viruses was low, from 45.6% nt (33.4% aa; OROV) to 55.0% nt (47.9% aa; Sango virus), which indicates that its M segment belongs to another virus, as previously suggested (*8*). On the other hand, the high sequence identity of all SBV genes to SATV and DOUV indicates that SBV belongs to the species *Sathuperi virus*.

**Table 2:** Pairwise comparison of full N, M, and L gene sequences of SBV with other orthobunyaviruses*

Species	SBV	*Shamonda virus*				*Sathuperi virus*			*Akabane virus*			*Shuni virus*			*Simbu virus*
		SHAV	PEAV	SANV		SATV	DOUV		AKAV	SABOV		SHUV	AINOV		SIMV
Small segment															
SHAV	**97.7 (100.0)**														
PEAV	77.2 (80.6)	77.5 (80.6)													
SANV	78.2 (80.6)	77.3 (80.6)	92.5 (100)												
SATV	**93.1 (99.5)**	92.9 (99.5)	77.6 (81.1)	78.1 (81.1)											
DOUV	**92.2 (99.1)**	92.9 (99.1)	77.8 (81.1)	78.3 (81.1)		92.9 (99.5)									
AKAV	77.2 (79.8)	77.3 (79.8)	76.8 (82.8)	77.6 (82.8)		78.6 (80.2)	78.2 (80.2)								
SABOV	78.6 (81.5)	78.9 (81.5)	76.6 (84.1)	76.5 (84.1)		78.9 (81.9)	79.9 (81.9)		81.2 (88.4)						
SHUV	77.5 (80.2)	77.5 (80.2)	91.8 (99.5)	90.8 (99.5)		77.6 (80.6)	78.3 (80.6)		76.9 (82.4)	77.5 (83.6)					
AINOV	76.9 (79.8)	76.6 (79.8)	91.2 (99.1)	91.2 (99.1)		78.2 (80.2)	78.3 (80.2)		77.3 (82.8)	78.1 (84.1)		93.9 (99.5)			
SIMV	78.6 (80.2)	78.6 (80.2)	77.8 (83.2)	77.5 (83.2)		79.2 (80.2)	80.5 (80.6)		78.8 (81.5)	77.8 (81.1)		78.1 (82.8)	78.5 (83.2)		
OROV	69.8 (69.9)	69.5 (69.9)	67.3 (69.5)	69.8 (69.5)		68.5 (69.9)	70.2 (69.5)		69.5 (68.2)	68.2 (69)		67.8 (69)	68 (69)		69.3 (72.1)
Medium segment															
SHAV	**48.2 (36.5)**														
PEAV	48.3 (36.6)	54.4 (47.6)													
SANV	48.1 (36.9)	55.0 (47.9)	83.9 (89.9)												
SATV	**82.1 (90.1)**	48.5 (36.7)	48.6 (37.2)	49.4 (37.3)											
DOUV	**81.7 (88.9)**	48.6 (36.6)	48.7 (37.0)	49.5 (36.9)		85.8 (91.5)									
AKAV	47.3 (35.6)	54.2 (48.6)	55.2 (49.4)	55.9 (49.2)		48.0 (35.8)	47.2 (36.3)								
SABOV	47.8 (36.8)	53.0 (46.8)	55.1 (48.2)	55.3 (49.6)		47.8 (36.7)	47.3 (36.6)		60.0 (56.4)						
SHUV	59.9 (56.0)	48.1 (35.6)	48.5 (35.9)	49.9 (36.8)		60.1 (56.3)	60.2 (56.3)		47.1 (36.0)	47.1 (34.8)					
AINOV	60.2 (56.9)	47.5 (35.0)	47.5 (35.7)	48.4 (36.5)		60.4 (57.1)	60.4 (57.0)		46.2 (35.1)	47.3 (35.4)		71.5 (76.1)			
SIMV	56.5 (50.8)	48.7 (37.7)	47.8 (37.2)	48.2 (37.5)		57.1 (50.1)	56.5 (49.8)		46.5 (37.3)	47.3 (36.7)		58.3 (50.1)	58.4 (51.4)		
OROV	48.6 (34.2)	45.6 (33.4)	46.3 (34.5)	45.8 (34.3)		48.1 (34.9)	47.7 (34.2)		44.8 (32.7)	45.0 (34.3)		48.9 (35.7)	48.9 (36.4)		50.2 (36.6)
Large segment															
SHAV	**92.9 (98.4)**														
PEAV	66.9 (68.7)	65.9 (68.3)													
SANV	66.7 (69.2)	66.5 (69.0)	84.8 (93.8)												
SATV	**84.5 (95.8)**	84.9 (95.7)	66.2 (68.1)	66.6 (68.6)											
DOUV	**84.5 (94.8)**	84.5 (94.4)	66.0 (68.1)	66.0 (68.5)		87.1 (96.1)									
AKAV	67.3 (70.8)	67.0 (70.8)	65.9 (68.2)	65.7 (68.1)		67.3 (70.9)	66.9 (70.3)								
SABOV	66.6 (70.7)	66.0 (70.7)	65.4 (67.9)	65.0 (68.0)		66.2 (70.4)	66.3 (70.2)		71.1 (78.9)						
SHUV	66.1 (68.6)	65.9 (68.3)	78.3 (88.8)	78.6 (90.5)		66.2 (68.4)	66.5 (68.2)		65.6 (68.1)	65.5 (68.0)					
AINOV	66.6 (68.7)	65.8 (68.5)	78.5 (88.5)	79.1 (90.1)		66.2 (68.2)	66.1 (68.2)		66.2 (67.9)	65.8 (67.8)		85.4 (95.4)			
SIMV	67.6 (69.9)	67.3 (69.7)	67.4 (70.7)	68.0 (70.7)		67.4 (70.3)	67.5 (70.0)		67.3 (70.1)	66.9 (70.4)		67.5 (70.8)	67.9 (71.0)		
OROV	60.4 (57.5)	60.0 (57.4)	61.2 (58.7)	60.9 (58.5)		60.1 (57.9)	60.5 (57.5)		60.9 (58.3)	60.1 (58.2)		61.3 (59.1)	61.1 (58.7)		60.7 (58.3)

*Values shown are nt (aa) identities, %. **Boldface** indicates SBV sequence identities compared with DOUV, SATV, and SHAV. SBV, Schmallenberg virus; SHAV, Shamonda virus; PEAV, Peaton virus; SANV, Sango virus; SATV, Sathuperi virus; DOUV, Douglas virus; AKAV, Akabane virus; SHUV, Shuni virus; AINOV, Aino virus; SIMV, Simbu virus.

A phylogenetic tree derived from the M gene (Figure, panel A) demonstrates that SBV and DOUV cluster closely with SATV, whereas SHAV was placed distantly from all other viruses. In contrast, phylogenetic trees of the L (Figure, panel B) and N (Figure, panel C) genes showed a close relationship between SBV and SHAV. Results for all genome segments show that SBV should be classified within the species *Sathuperi virus* and is likely to be the ancestor of SHAV, which is in contrast a reassortant virus comprising the S and L segments from SBV and the M segment from another virus, as proposed previously (*8*).

**Figure:**
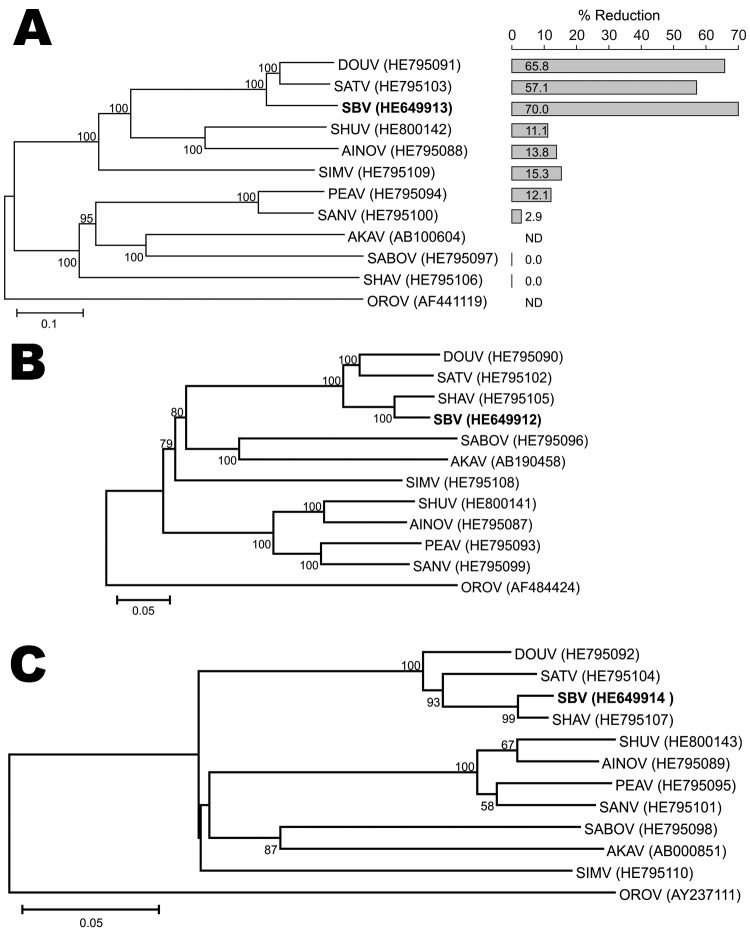
Maximum-likelihood trees showing phylogenetic relationships of Simbu serogroup viruses for the M (A), L (B), and S (C) coding regions. The bar plot in panel A indicates the percentage of titer reduction of each virus by anti–Schmallenberg virus serum. GenBank accession numbers are shown. Numbers at nodes indicate percentage of 1,000 bootstrap replicates (values <50 are not shown). Scale bars indicate nucleotide substitutions per site. DOUV, Douglas virus; SATV, Sathuperi virus; SBV, Schmallenberg virus; SHUV, Shuni virus; AINOV, Aino virus; SIMV, Simbu virus; PEAV, Peaton virus; SANV, Sango virus; AKAV, Akabane virus; SABOV, Sabo virus; SHAV, Shamonda virus; OROV, Oropouche virus. ND, not determined.

To further clarify the placement of SBV within the Simbu serogroup, we determined the ability of SBV antibodies to neutralize other Simbu serogroup viruses. We performed titer reduction assays of the 9 other viruses with anti-SBV serum; the viruses were propagated in BHK-21 cells clone CT (L164, Collection of Cell Lines in Veterinary Medicine, Friedrich-Loeffler-Institute, Riems, Germany). Neutralizing potencies of SBV antibodies against Simbu serogroup virus strains were tested by titrating virus in the presence of anti-SBV serum (neutralizing titer 32); results were expressed as percentage titer reduction in relation to a parallel test without antiserum. Although DOUV and SATV were well neutralized, SHAV titers were not reduced at all (Figure, panel A), which supports the M gene phylogeny. Thus, phylogeny and cross-reactivity identified SHAV, but not SBV, as a reassortant within the Simbu serogroup.

## Conclusions

Although our results do not support the suggestions of Yanase et al. (*9*), they are fully consistent with the conclusions of Saeed et al. (*8*). On the basis of our results and those of Saeed et al. (*8*), we suggest that SHAV should be reclassified into the species *Sathuperi virus* and that the species *Shamonda virus* should be renamed *Peaton virus* or *Sango virus*.

In addition to showing that SBV belongs to the species *Sathuperi virus*, our results show that the virus is most likely not a reassortant and is likely to be one of the ancestors of SHAV, whereas SHAV is a reassortant comprising the SBV S and L genomic segments and the M segment from an unclassified virus. These detailed insights into the phylogeny of SBV could be the basis for the development of efficient, cross-protective vaccines. Our results also highlight the importance of full-genome analyses to identify potential genetic reassortments and to investigate the evolutionary history of viruses with segmented genomes.
